# Changes of functional brain network topology associated with nutritional indicator of patients with recurrent major depressive disorder

**DOI:** 10.3389/fnut.2025.1615978

**Published:** 2025-06-18

**Authors:** Guanghui Liu, Yanli Jiang, Chunpeng Zhou, Xiangxiang Wang, Min Dai, Jinping Zhang, Jing Yu, Gangzhong Zhang

**Affiliations:** ^1^Department of Neurosurgery, Zhengzhou Seventh People’s Hospital, Zhengzhou, China; ^2^Department of Radiology, Children’s Hospital of Nanjing Medical University, Nanjing, China; ^3^Department of Neurology, Zhengzhou Seventh People’s Hospital, Zhengzhou, China; ^4^Department of Radiology, Nanjing Brain Hospital, Affiliated Hospital of Nanjing Medical University, Nanjing, China

**Keywords:** major depressive disorder, resting-state functional magnetic resonance imaging, functional brain network, recurrence, brain-derived neurotrophic factor

## Abstract

**Introduction:**

Major depressive disorder (MDD) is generally categorized into first-episode MDD (fMDD) and recurrent MDD (rMDD). This study aimed to investigate the changes of brain network, as well as the relationships between relapses, brain regions, nutritional and metabolic indicators by resting-state functional magnetic resonance imaging (rs-fMRI) and graph theoretical analysis.

**Methods:**

Thirty-two fMDD patients, 32 rMDD patients, and 32 healthy controls (HCs) underwent rs-fMRI scanning. Graph theoretical analysis was applied to examine the nodal strength and nodal global efficiency of brain networks. In addition, the nutritional and metabolic indicators were acquired from all patients. The differences of demographic, clinical data and topological parameters between groups were compared. Moreover, the relationships between number of relapses, topological parameters of brain regions, nutritional and metabolic indicators were evaluated.

**Results:**

Patients with rMDD showed decreased level of brain-derived neurotrophic factor (BDNF) when compared with those with fMDD. Both fMDD and rMDD patients showed decreased nodal strength and global efficiency in the left amygdala compared to HCs. Additionally, rMDD patients exhibited more extensive network disruptions, including decreased nodal strength in the right middle frontal gyrus, left middle cingulate gyrus, left posterior cingulate gyrus, left hippocampus, and right amygdala, as well as decreased nodal global efficiency in multiple regions involved in emotional processing and cognitive control. Moreover, the number of episodes were negatively associated with the level of BDNF, nodal strength of right amygdala and nodal global efficiency of right amygdala of rMDD patients. The level of BDNF were positively related to nodal strength of right amygdala and nodal global efficiency of right amygdala of rMDD patients.

**Conclusion:**

Our findings revealed distinct patterns of brain network topology between fMDD and rMDD patients, with rMDD patients showing more widespread disruptions, which might be associated with greater number of relapses and worse level of neurological nutrition. These results suggested that recurrent depressive episodes might related to progressive disruptions in brain, particularly in regions involved in emotional processing and regulation.

## 1 Introduction

Major depressive disorder (MDD) represents one of the most prevalent and debilitating psychiatric conditions worldwide, characterized by persistent feelings of sadness, anhedonia, and various cognitive, emotional, and physical symptoms (1–3). Recent epidemiological studies indicate that MDD affects more than 264 million people globally, with a lifetime prevalence ranging from 3 to 21% in most developed countries (2, 4). The socioeconomic burden of MDD is substantial, with annual costs exceeding $326 billion in direct healthcare expenses and indirect costs such as lost productivity (5). The World Health Organization projects that by 2030, MDD will become the leading cause of disease burden globally, surpassing cardiovascular diseases and cancer (6). Moreover, approximately 35% of patients show inadequate response to first-line treatments, and up to 30% develop treatment-resistant depression, highlighting the urgent need for better understanding of disease mechanisms and more targeted therapeutic approaches (2, 7).

The clinical heterogeneity of MDD poses significant challenges for both research and treatment. MDD typically manifests in two distinct presentations: first-episode MDD (fMDD), representing the initial occurrence of depressive symptoms, and recurrent MDD (rMDD), characterized by multiple depressive episodes throughout the patient’s lifetime (8). The progression from first-episode to recurrent depression represents a critical transition in the disease course, with studies showing that approximately 60% of patients who recover from their first-episode experience recurrence, and this risk escalates to 90% after two episodes (9–11). This high recurrence rate suggests fundamental changes in brain function between first-episode and recurrent depression (12). Furthermore, each subsequent episode appears to increase the likelihood of future recurrences and may be associated with progressive cognitive decline and functional impairment. Despite the clinical significance of this progression, the underlying neural mechanisms driving the transition from first-episode to recurrent depression remain poorly understood (13, 14).

Recent advances in neuroscience have revolutionized our understanding of MDD pathophysiology, revealing that it extends far beyond simple monoamine deficiency to encompass complex alterations in neural circuits, neuroplasticity, and network dynamics (15–17). The emergence of network neuroscience has provided compelling evidence that psychiatric disorders, including MDD, can be conceptualized as disorders of brain network organization rather than isolated regional dysfunction (18). This network perspective has particular relevance for understanding the progression from fMDD to rMDD, as it may help explain how initial localized disruptions could propagate through neural circuits to create more widespread dysfunction over time (15, 18, 19). The network approach also aligns with the complex symptomatology of MDD, which includes disturbances in emotion, cognition, motivation, and physiological functions, suggesting dysfunction across multiple interacting neural systems rather than isolated brain regions.

Previous neuroimaging studies have identified widespread alterations in both structural and functional brain networks in MDD patients. These changes particularly affect regions involved in emotion processing (such as the amygdala and anterior cingulate cortex), cognitive control (such as the dorsolateral prefrontal cortex), and self-referential processing (such as the default mode network) (20–22). The limbic system, particularly the amygdala, has emerged as a critical hub in depression pathophysiology, showing consistent alterations across multiple studies (23). Neuroimaging studies have demonstrated that amygdala dysfunction correlates with symptom severity and treatment response, suggesting its potential role as a biomarker for disease progression. Additionally, altered connectivity between the amygdala and prefrontal regions has been implicated in emotional dysregulation, a core feature of MDD. However, most research has examined either fMDD or rMDD in isolation, leaving a significant gap in our understanding of how brain network organization differs between these two disease stages (24, 25).

The amygdala related pathways involved in emotion regulation mainly include the following (26–28): (1) thalamus-amygdala pathway, known as the short pathway: sensory information is transmitted from sensory organs to the thalamus, and is directly projected to the amygdala without going through fine processing in the cerebral cortex. (2) The cerebral cortex-thalamus-amygdala pathway, also known as the long pathway: sensory information is first transmitted from sensory organs to the thalamus, which then transmits the information to the cerebral cortex for detailed processing. (3) The amygdala-hippocampus pathway, which plays an important role in emotional learning and memory. The hippocampus also participates in the formation and storage of memory.

The occurrence of depression is also related to disturbances in nutrition of the central nervous system, as well as abnormal peripheral glucose and lipid metabolism (29). Brain neurogenesis is involved in the occurrence of depression through brain-derived neurotrophic factor (BDNF), an important neurotransmitter regulator (30). In the process of neurogenesis in depression, there are multiple regulatory signaling pathways, including the BDNF–TrkB signaling pathway. PI3K and Akt play important roles in neurogenesis under the action of BDNF. Almost all antidepressant drugs can enhance TrkB phosphorylation, activate this pathway to promote neurogenesis, and TrkB antagonists can block the antidepressant effect of drugs.

The purpose of this study was to investigate the differences in functional brain network topology between patients with fMDD and rMDD, and their relationships with nutritional, metabolic indicators using resting-state functional magnetic resonance imaging (rs-fMRI) and graph theory analysis. By examining network properties across the whole brain, we aimed to identify specific regional differences in nodal strength and global efficiency between these two patient groups and healthy controls (HCs). We hypothesized that both fMDD and rMDD patients would show altered network properties compared to HCs, and that rMDD patients might demonstrate more extensive network disruptions compared to fMDD patients, reflecting the cumulative impact of multiple depressive episodes on brain network organization. In addition, we hypothesized that the number of relapses might be associated with abnormal brain regions, impaired nutritional and metabolic indicators. Understanding the distinct patterns of brain network organization in fMDD versus rMDD might provide insights into the underlying mechanisms of disease progression and potentially guide the development of more targeted therapeutic strategies (such as neural regulation targeting bilateral amygdala or dorsolateral prefrontal cortex) for different stages of MDD. This knowledge could be particularly valuable for early intervention and prevention of recurrence in MDD patients, ultimately leading to improved treatment outcomes.

## 2 Materials and methods

### 2.1 Participants

This study was approved by the Medical Research Ethics Committee of Zhengzhou Seventh People’s Hospital and Nanjing Brain Hospital. Moreover, written informed consent was acquired from all subjects. A total of 32 fMDD patients and 32 rMDD patients were recruited. In addition, 32 right-handed HCs matched for age, gender and educational level were recruited.

The inclusion criteria were as follows: (1) 20–45 years of age; (2) at least 9 years of education; (3) right-handed; (4) Han Chinese. The diagnosis of MDD was made by two experienced psychiatrists according to the Diagnostic and Statistical Manual of Mental Disorders-fifth Edition (DSM-5) criteria. Both fMDD and rMDD patients had total scores of 17-item Hamilton Depression Rating Scale (HAMD-17) more than 17. The fMDD patients had no previous medication history. In addition, all HCs had total scores of HAMD-17 less than 7, and were screened through diagnostic interviews with the Structured Clinical Interview for DSM-5 Nonpatient Edition by two experienced psychiatrists, to exclude any current or history of psychiatric disorders.

The exclusion criteria were as follows: (1) uncontrolled or serious physical illness; (2) any other psychiatric, neurological, or somatic disorders; (3) history of any antidepressant medication 2 weeks before MRI scan; (4) history of head trauma or structural brain abnormalities identified by routine MRI; (5) abuse of alcohol or drugs; (6) female who were regnant, lactating or menstruating; (7) any contraindication for MRI scan.

The metabolic indicators including fasting blood glucose (FPG), glycated hemoglobin (HbA1c), total cholesterol (TC), triglycerides (TG), high-density lipoproteins (HDL) and low-density lipoproteins (LDL), as well as the nutritional indicators including BDNF and nerve growth factor (NGF), were acquired from all patients.

### 2.2 MRI data acquisition

MRI images were acquired using a 3.0 Tesla scanner (GE). All subjects were instructed to keep eyes closed, stay relaxed and awake, think of nothing in particular during MRI scanning. T1-weighted structural images were acquired with the following parameters: repetition time (TR) = 7.7 ms; echo time (TE) = 3.1 ms; slice thickness = 1 mm; field of view (FOV) = 256 × 256 mm^2^; matrix = 256 × 256; flip angle (FA) = 12°; slices = 160. The rs-MRI images were obtained with the following parameters: TR = 2000 ms; TE = 30 ms; slice thickness = 3.5 mm; FOV = 224 × 224 mm^2^; matrix = 80 × 80; FA = 90°; slices = 33; volumes = 240. Participants were excluded if structural abnormalities or artifacts were identified by structural MRI scans.

### 2.3 MRI data preprocessing

MRI data were preprocessed using the software of Data Processing Assistant for Resting-State fMRI (DPARSF) (31). The main steps were as follows: (1) removal of the first 10 time points; (2) slice timing; (3) head motion correction and subjects with translational motion exceeding 2 mm or rotational motion exceeding 2° were excluded; (4) spatial normalization into the standard Montreal Neurological Institute (MNI) space and re-sample into a voxel size of 3 mm × 3 mm × 3 mm; (5) spatial smoothing with a Gaussian kernel of 4 mm × 4 mm × 4 mm; (6) linear detrending; (7) temporal band-pass filtering at a frequency band of 0.01–0.1 Hz; (8) nuisance covariates regression (the head motion parameters, white matter and cerebrospinal fluid signals).

### 2.4 Construction of functional brain network

The graph theory analysis method defines a single brain region as a ‘node’ and defines the functional connectivity between brain regions as ‘edge’, simplifies the entire brain into a network diagram composed of nodes and edges. Firstly, the whole brain was divided into 90 nodes using the AAL template (including cortical and subcortical regions, excluding cerebellar regions) provided by the Montreal Institute (MNI) (32). Secondly, the *Pearson* correlation coefficients of the average time series between each pair of 90 brain regions were calculated to define the network edges.

### 2.5 Calculation of nodal topological parameters

Based on the GRETNA software package (33), the nodal topological parameters including nodal strength and nodal global efficiency were calculated (34). The sparsity was selected as 0.05–0.5, with an interval of 0.05. The area under curve (AUC) of these two metrics were calculated over all sparsity range.

### 2.6 Statistical analysis

The differences of demographic and clinical characteristics between patients and HCs were evaluated by one-way analysis of variance (ANOVA) and *post hoc* contrasts with the method of least-significant difference (LSD) (age and educational level), two-sample *t*-tests (HAMD sores, age of onset, metabolic and nutritional indicators), and *Pearson* chi-square test (gender). The statistical significances were set at *p* < 0.05.

In addition, the differences of nodal topological parameters including nodal strength and nodal global efficiency between patients and HCs were evaluated by ANOVA and *post hoc* contrasts with the method of LSD. The statistical significances were set at *p* < 0.05, and the multiple comparisons were corrected using the family-wise error (FWE) method with Bonferroni-corrected significance thresholds.

Moreover, the relationships between number of relapses, topological parameters of brain regions, nutritional and metabolic indicators were evaluated. The statistical significances were set at *p* < 0.05.

## 3 Results

### 3.1 Comparisons of demographic and clinical characteristics between patients and HCs

There were no significant differences in the gender, age and educational level among groups of fMDD, rMDD patients and HCs. In addition, no significant differences were found in the HAMD-17 scores, age of onset, metabolic indicators (FPG, HbA1c, TC, TG, HDL, LDL) and NGF level between fMDD and rMDD patients. However, patients with rMDD showed increased illness duration and decreased level of BDNF when compared with those with fMDD (Table 1).

**Table 1:** The demographic and clinical characteristics of fMDD, rMDD patients and HCs.

Characteristics	fMDD	rMDD	HCs	*χ^2^*/*F*/*t*	*p*
Gender (male/female)	15/17	15/17	14/18	0.084	0.96
Age (years)	33.06 ± 7.16	34.06 ± 7.59	31.47 ± 7.98	0.95	0.39
Educational level (years)	13.66 ± 2.75	13.84 ± 2.52	13.19 ± 2.71	0.52	0.60
HAMD-17 (scores)	25.09 ± 4.92	26.47 ± 5.61	-	−1.04	0.30
Age of onset (years)	33.06 ± 7.16	32.19 ± 7.38	-	0.48	0.63
Number of episodes	-	2.94 ± 0.84	-	-	-
Illness duration (months)	3.84 ± 1.14	13.47 ± 5.04	-	−10.55	<0.01
Interval between episodes (months)	-	7.69 ± 3.91	-	-	-
Metabolic indicators					
FPG (mmol/L)	5.08 ± 0.71	5.03 ± 0.69	-	0.24	0.81
HbA1c (%)	5.12 ± 0.57	4.97 ± 0.63	-	1.03	0.31
TC (mmol/L)	4.06 ± 0.76	3.94 ± 0.81	-	0.62	0.54
TG (mmol/L)	1.08 ± 0.34	1.10 ± 0.37	-	−0.20	0.85
HDL (mmol/L)	1.32 ± 0.12	1.36 ± 0.12	-	−1.19	0.24
LDL (mmol/L)	2.61 ± 0.31	1.36 ± 0.32	-	1.23	0.23
Nutritional biomarkers					
BDNF (ng/mL)	30.15 ± 4.88	26.97 ± 5.79	-	2.37	0.02
NGF (ng/L)	9.49 ± 3.30	8.68 ± 2.93	-	1.03	0.31

MDD, major depressive disorder; fMDD, first-episode MDD patients; rMDD, recurrent MDD patients; HCs, healthy controls; HAMD-17, 17-item Hamilton Depression Rating Scale; FPG, fasting blood glucose; HbA1c, glycated hemoglobin; TC, total cholesterol; TG: triglycerides; HDL, high-density lipoproteins; LDL, low-density lipoproteins; BDNF, brain-derived neurotrophic factor; NGF, nerve growth factor. p values were obtained by one-way analysis of variance (ANOVA) and post hoc contrasts with the method of least-significant difference (LSD) (age and educational level), two-sample *t*-tests (HAMD sores, age of onset, metabolic and nutritional indicators), and Pearson chi-square test (gender). The statistical significances were set at *p* < 0.05.

### 3.2 Comparisons of nodal topological parameters between patients and HCs

**Figure 1:**
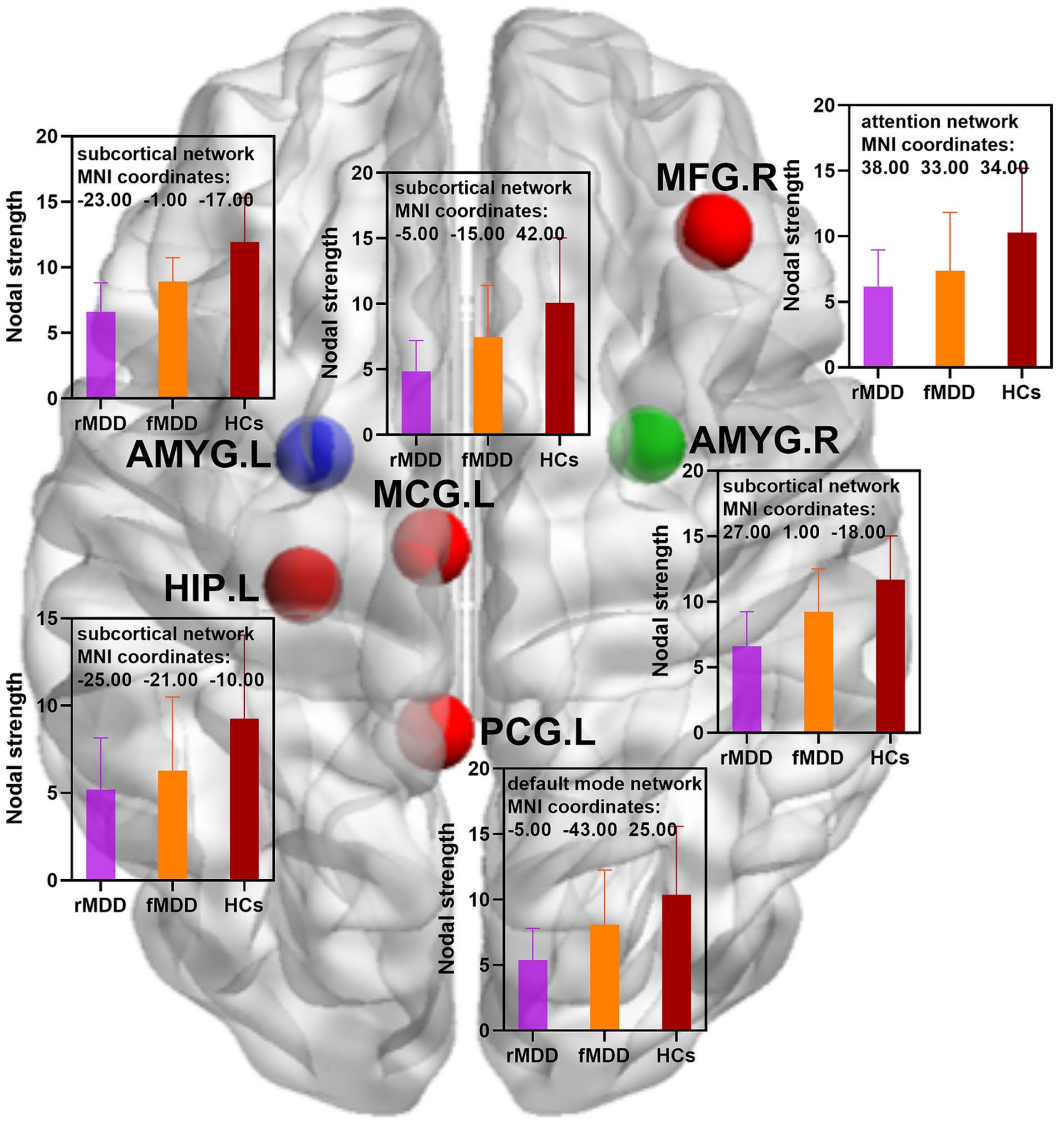
Changed nodal strength of functional brain networks in fMDD and rMDD patients. MDD, major depressive disorder; fMDD, first-episode MDD patients; rMDD, recurrent MDD patients; MFG.R, right middle frontal gyrus; MCG.L, left middle cingulate gyrus; PCG.L, left posterior cingulate gyrus; HIP.L, left hippocampus; AMYG.L, left amygdala; AMYG.R, right amygdala. Red nodes indicated decreased nodal strength in rMDD patients when compared with HCs; blue nodes indicated decreased nodal strength in both fMDD and rMDD when compared with HCs; green node indicated decreased nodal strength in rMDD patients when compared with fMDD patients. MIN, Montreal Institute of Neurology. *p* values were obtained by one-way analysis of variance (ANOVA) and *post hoc* contrasts with the method of least-significant difference (LSD). The statistical significances were set at *p* < 0.05, and the multiple comparisons were corrected using the family-wise error (FWE) method with Bonferroni-corrected significance thresholds.

**Figure 2:**
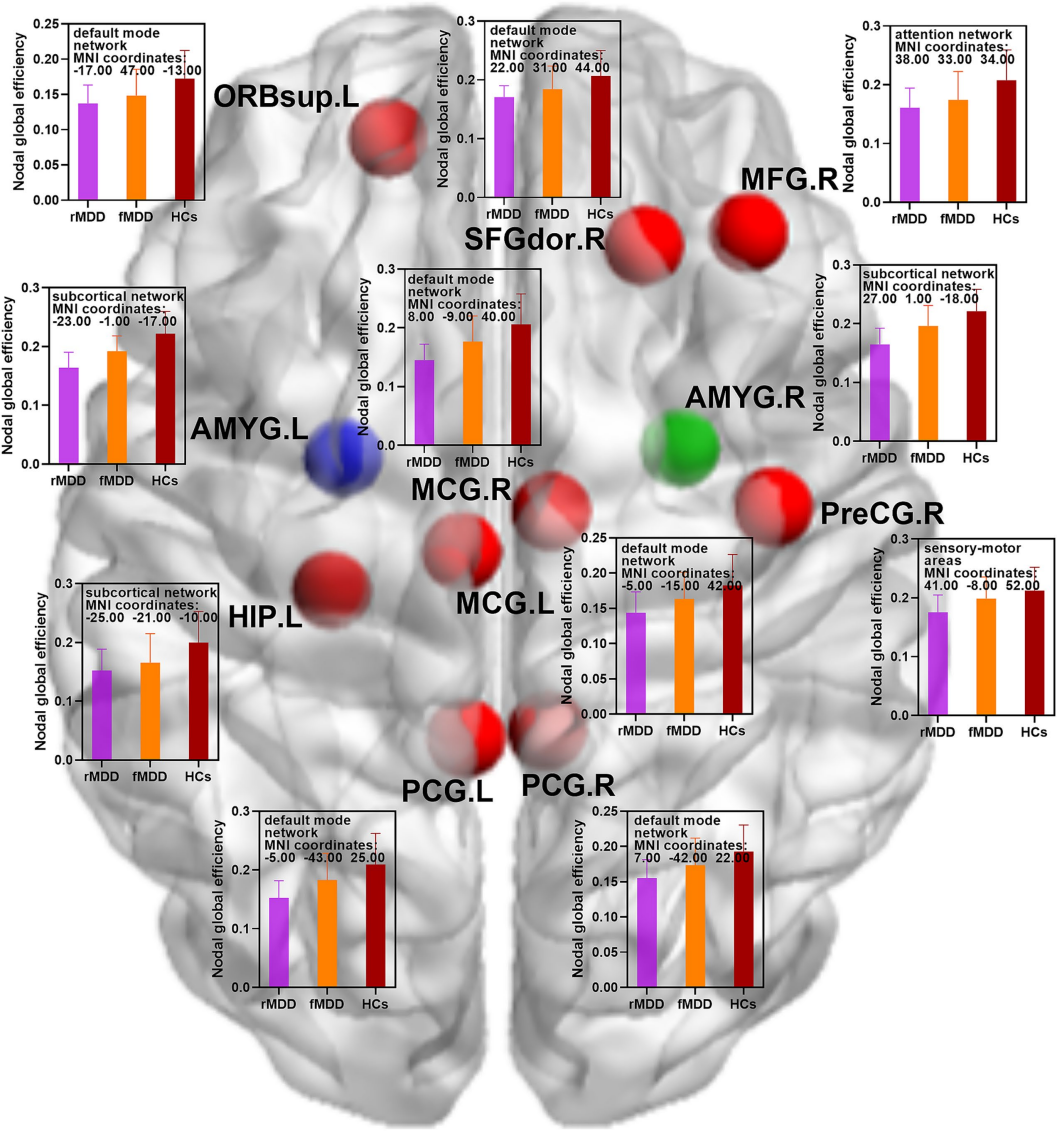
Changed nodal global efficiency of functional brain networks in fMDD and rMDD patients. MDD, major depressive disorder; fMDD, first-episode MDD patients; rMDD, recurrent MDD patients; PreCG.R, right precental gyrus; SFGdor.R, right dorsolateral superior frontal gyrus; ORBsup.L, left orbital part of superior frontal gyrus; MFG.R, right middle frontal gyrus; MCG.L, left middle cingulate gyrus; MCG.R, right middle cingulate gyrus; PCG.L, left posterior cingulate gyrus; PCG.R, right posterior cingulate gyrus; HIP.L, left hippocampus; AMYG.L, left amygdala; AMYG.R, right amygdala. Red nodes indicated decreased nodal global efficiency in rMDD patients when compared with HCs; blue nodes indicated decreased nodal global efficiency in both fMDD and rMDD when compared with HCs; green node indicated decreased nodal global efficiency in rMDD patients when compared with fMDD patients. MIN, Montreal Institute of Neurology. *p* values were obtained by one-way analysis of variance (ANOVA) and post hoc contrasts with the method of least-significant difference (LSD). The statistical significances were set at *p* < 0.05, and the multiple comparisons were corrected using the family-wise error (FWE) method with Bonferroni-corrected significance thresholds.

Compared with HCs, rMDD patients showed decreased nodal strength in the right middle frontal gyrus, left middle cingulate gyrus, left posterior cingulate gyrus, left hippocampus, left amygdala and right amygdala. In addition, rMDD patients exhibited decreased nodal global efficiency in the right precental gyrus, right dorsolateral superior frontal gyrus, left orbital part of superior frontal gyrus, right middle frontal gyrus, left middle cingulate gyrus, right middle cingulate gyrus, left posterior cingulate gyrus, right posterior cingulate gyrus, left hippocampus, left amygdala and right amygdala (Table 2; Figures 1, 2).

**Table 2:** Changed nodal topological parameters of functional brain networks in fMDD and rMDD patients.

Brain regions	fMDD	rMDD	HCs	*F*	*p*
Nodal strength					
Right middle frontal gyrus	6.16 ± 2.79	7.37 ± 4.45	10.29 ± 4.90	8.37	0.00045^a^
Left middle cingulate gyrus	4.86 ± 2.34	7.48 ± 3.93	10.09 ± 4.95	14.45	0.000003^a^
Left posterior cingulate gyrus	5.39 ± 2.41	8.08 ± 4.17	10.37 ± 5.23	11.80	0.000027^a^
Left hippocampus	5.19 ± 2.97	6.28 ± 4.21	9.26 ± 4.77	8.64	0.00036^a^
Left amygdala	6.61 ± 2.21	8.93 ± 1.81	11.95 ± 3.36	35.38	3.75E-12^a,b,c^
Right amygdala	6.61 ± 2.63	9.25 ± 3.27	11.69 ± 3.33	21.65	1.91E-8^a,c^
Nodal global efficiency					
Right precental gyrus	0.18 ± 0.03	0.20 ± 0.04	0.21 ± 0.04	8.78	0.00032^a^
Right dorsolateral superior frontal gyrus	0.17 ± 0.02	0.18 ± 0.04	0.21 ± 0.04	8.49	0.00041^a^
Left orbital part of superior frontal gyrus	0.14 ± 0.03	0.15 ± 0.04	0.17 ± 0.04	8.58	0.00038^a^
Right middle frontal gyrus	0.16 ± 0.03	0.17 ± 0.05	0.21 ± 0.05	9.08	0.00025^a^
Left middle cingulate gyrus	0.15 ± 0.03	0.18 ± 0.04	0.21 ± 0.05	16.68	6.47E-7^a^
Right middle cingulate gyrus	0.14 ± 0.03	0.16 ± 0.04	0.18 ± 0.04	8.57	0.00038^a^
Left posterior cingulate gyrus	0.15 ± 0.03	0.18 ± 0.05	0.21 ± 0.05	13.73	0.000006^a^
Right posterior cingulate gyrus	0.16 ± 0.03	0.17 ± 0.04	0.19 ± 0.04	9.52	0.00017^a^
Left hippocampus	0.15 ± 0.04	0.17 ± 0.05	0.20 ± 0.05	8.77	0.00032^a^
Left amygdala	0.16 ± 0.03	0.19 ± 0.03	0.22 ± 0.04	29.23	1.42E-10^a,b,c^
Right amygdala	0.16 ± 0.03	0.20 ± 0.04	0.22 ± 0.04	22.99	7.70E-9^a,c^

MDD, major depressive disorder; fMDD, first-episode MDD patients; rMDD, recurrent MDD patients; HCs, healthy controls. ^a^decreased nodal topological parameters in rMDD patients when compared with HCs; ^b^decreased nodal topological parameters in fMDD patients when compared with HCs; ^c^decreased nodal topological parameters in rMDD patients when compared with fMDD patients. *p* values were obtained by one-way analysis of variance (ANOVA) and post hoc contrasts with the method of least-significant difference (LSD). The statistical significances were set at P < 0.05, and the multiple comparisons were corrected using the family-wise error (FWE) method with Bonferroni-corrected significance thresholds.

Compared with HCs, fMDD patients demonstrated decreased nodal strength in the left amygdala and decreased nodal global efficiency in the left amygdala (Table 2; Figures 1, 2).

Compared with fMDD patients, rMDD patients demonstrated decreased nodal strength in the right amygdala and decreased nodal global efficiency in the right amygdala (Table 2; Figures 1, 2).

### 3.3 Relationships between abnormal brain regions and clinical characteristics

**Figure 3:**
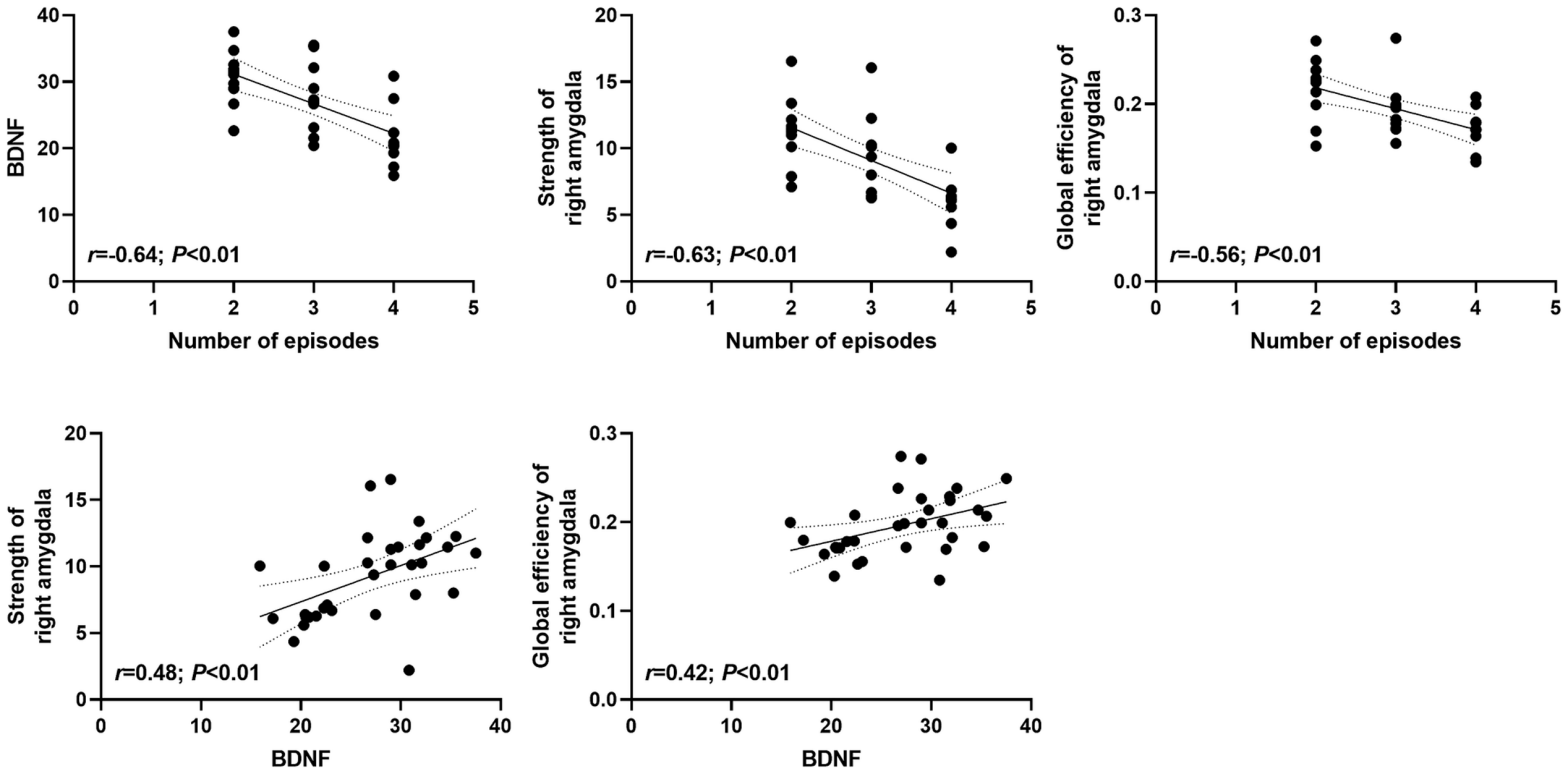
Relationships between abnormal brain regions and clinical characteristics. BDNF, brain-derived neurotrophic factor. *p* values were obtained by *Pearson* correlation analysis.

The number of episodes of MDD were negatively associated with the level of BDNF (*r* = −0.64; *p* < 0.01), nodal strength of the right amygdala (*r* = −0.63; *p* < 0.01) and nodal global efficiency of the right amygdala (*r* = −0.56; *p* < 0.01) of rMDD patients. In addition, the level of BDNF were positively related to the nodal strength of the right amygdala (*r* = 0.48; *p* < 0.01) and nodal global efficiency of the right amygdala (*r* = 0.42; *p* < 0.01) in rMDD patients (Figure 3).

## 4 Discussion

In this study, we investigated the differences in functional brain network topology between patients with fMDD and rMDD using rs-fMRI and graph theory analysis. Our findings revealed that both fMDD and rMDD patients exhibited altered brain network properties when compared to HCs, with rMDD patients showing more extensive disruptions in network organization, particularly in the amygdala and other emotion-related regions. In addition, the number of episodes were negatively associated with BDNF level, nodal parameters of right amygdala while BDNF level were positively related to nodal parameters of right amygdala in rMDD patients. These findings suggested that rMDD patients had more widespread disruptions, which might be related to greater number of relapses and worse level of neurological nutrition. All these demonstrated that that recurrent depressive episodes might related to progressive disruptions in brain.

Firstly, in comparison with HCs, both fMDD and rMDD patients showed decreased nodal strength and global efficiency in the left amygdala (the affected side of the amygdala), a critical region in emotional processing (35). The decreased nodal strength indicated reduced functional connectivity between the amygdala and other brain regions, while reduced global efficiency suggested compromised information processing capability within the amygdala-centered network (36, 37). These alterations aligned with previous studies demonstrating abnormal amygdala function in MDD, suggesting that disrupted amygdala connectivity might be a core feature of depression regardless of episode status (38, 39).

Notably, rMDD patients exhibited more extensive network disruptions compared to both HCs and fMDD patients. Beyond the amygdala, rMDD patients showed decreased nodal strength in multiple regions including the right middle frontal gyrus, left middle cingulate gyrus, left posterior cingulate gyrus, and left hippocampus. These regions are key components of the emotional processing and cognitive control networks (40, 41). The middle frontal gyrus is involved in emotional regulation and executive function, while the cingulate cortex plays a crucial role in emotional awareness and cognitive control (40, 41). Additionally, the reduced global efficiency in the right precentral gyrus and right dorsolateral superior frontal gyrus suggested compromised top-down control circuits essential for both motor control and executive function (3). The decreased efficiency in the left orbital part of superior frontal gyrus, a region crucial for emotional processing and decision-making, further indicated disruption of frontal-limbic circuits that were fundamental for mood regulation (42). The bilateral involvement of middle and posterior cingulate regions indicated a more systemic disruption of the emotion regulation network, potentially explaining the increased difficulty in emotional regulation observed in rMDD patients (43). The hippocampus, working in concert with the amygdala, is essential for emotional memory processing (44). The widespread network disruptions in rMDD patients suggested that recurrent episodes might be associated with more pervasive alterations in brain network organization.

Our findings highlighted a particularly interesting hemispheric difference in amygdala dysfunction between fMDD and rMDD patients. While fMDD patients showed only left amygdala (the affected side of the amygdala) alterations, rMDD patients demonstrated bilateral amygdala abnormalities. This hemispheric difference might reflect the progression of network dysfunction from first-episode to recurrence. The bilateral nature of amygdala dysfunction in rMDD patients might explain their potentially higher risk of recurrence and more severe clinical presentations (45, 46). This pattern of progressive network disruption suggested that the cumulative impact of multiple depressive episodes might be associated with more severe disruptions in the brain’s emotional processing networks.

The reduced nodal global efficiency observed in rMDD patients across multiple regions suggested compromised network integration and information processing. These more extensive disruptions in rMDD patients indicated that the chronic nature of the illness might exacerbate these deficits, which might be associated with a more compromised brain network organization. This finding had important implications for understanding disease progression and treatment approaches, as it suggested that early intervention might be crucial in preventing the development of more widespread network dysfunction.

The ‘Neurotrophic Hypothesis of Depression’ suggests that the interruption of neurotrophic support is a key mechanism for MDD related synaptic and brain related functional changes (16). Neurotrophic factors are responsible for the formation, support, and plasticity of neural networks. Numerous studies have shown that the levels of neurotrophic factors in the blood are reduced in patients with persistent and recurrent depression, as well as in animal models of depression. In this study, BDNF level were negatively associated with the number of episodes, and positively related to nodal parameters of right amygdala in rMDD patients. These findings suggested that rMDD patients had worse level of neurological nutrition, which might related to progressive disruptions in brain.

These findings had several potential clinical implications. The distinct patterns of network disruption between fMDD and rMDD suggested that different therapeutic approaches might be needed for these two groups. For example, interventions targeting bilateral amygdala function might be more relevant for rMDD patients, while focused left amygdala (the affected side of the amygdala) interventions might be more appropriate for fMDD patients. Treatment strategies that addressed both the emotional and cognitive aspects of the disorder, such as cognitive behavioral therapy (CBT) or neuromodulation techniques like transcranial magnetic stimulation (TMS), might be particularly beneficial for patients with recurrent depression. These therapies could be tailored to target the specific brain regions with altered network properties, offering a more effective approach for managing both acute and recurrent depressive episodes (47–49).

Several limitations of this study should be noted. Firstly, the cross-sectional design prevents us from determining whether the observed network differences were causes or consequences of recurrence. Longitudinal studies were needed to clarify the temporal evolution of these network alterations. Secondly, while the current study focused on rs-fMRI (49), future research could incorporate task-based fMRI or other neuroimaging techniques such as diffusion tensor imaging (DTI) to further explore the structural connectivity changes that might underlie the observed functional disruptions. Finally, exploring the relationship between brain network alterations and clinical factors such as different symptoms and response to treatment would provide a more comprehensive understanding of the functional significance of these changes.

## 5 Conclusion

In conclusion, this study revealed distinct patterns of functional brain network topology between patients with fMDD and rMDD using rs-fMRI and graph theoretical analysis. Our findings demonstrated that both fMDD and rMDD patients showed altered network properties in the left amygdala (the affected side of the amygdala) compared to HCs. More importantly, rMDD patients exhibited more extensive network disruptions, including bilateral amygdala abnormalities and decreased nodal strength and global efficiency in multiple emotion-related brain regions. Greater number of relapses were associated with decreased BDNF level and impaired function of right amygdala in rMDD patients while decreased BDNF level were related to impaired function of right amygdala in rMDD patients. These findings suggested that recurrent depressive episodes might be associated with progressive disruptions in brain, particularly in regions involved in emotional processing and regulation, which provided important implications for early intervention and personalized treatment strategies in MDD patients.

## Data Availability

The raw data supporting the conclusions of this article will be made available by the authors, without undue reservation.
